# New species of *Cylindrocladiella* from plantation soils in South-East Asia

**DOI:** 10.3897/mycokeys.32.23754

**Published:** 2018-03-15

**Authors:** Nam Q. Pham, Irene Barnes, ShuaiFei Chen, Thu Q. Pham, Lorenzo Lombard, Pedro W. Crous, Michael J. Wingfield

**Affiliations:** 1 Department of Plant and Soil Sciences, Forestry and Agricultural Biotechnology Institute (FABI), University of Pretoria, Pretoria, South Africa; 2 Department of Department of Biochemistry, Genetics and Microbiology, Forestry and Agricultural Biotechnology Institute (FABI), University of Pretoria, Pretoria, South Africa; 3 China Eucalypt Research Centre (CERC), Chinese Academy of Forestry (CAF), Zhanjiang 524022, Guangdong Province, China; 4 Forest Protection Research Centre (FPRC), Vietnamese Academy of Forest Sciences (VAFS), 46 Duc Thang Road, Duc Thang Ward, Northern Tu Liem District, Hanoi 100000, Vietnam; 5 Westerdijk Fungal Biodiversity Institute, Uppsalalaan 8, 3584 CT Utrecht, The Netherlands

**Keywords:** multigene phylogeny, plantation forestry, taxonomy

## Abstract

*Cylindrocladiella* spp. are widely distributed especially in tropical and sub-tropical regions, where they are mainly known as saprobes although some species are plant pathogens. Very little is known about these fungi in South-East Asia. The aim of this study was to identify a collection of *Cylindrocladiella* isolates from soils collected in forest nurseries and plantations in Vietnam and Malaysia. This was achieved using DNA sequence comparisons and morphological observations. The study revealed two previously described species, *Cy. lageniformis* and *Cy. peruviana* as well as five novel taxa, described here as *Cy. arbusta* **sp. nov.**, *Cy. malesiana* **sp. nov.**, *Cy. obpyriformis* **sp. nov.**, *Cy. parvispora* **sp. nov.** and *Cy. solicola* **sp. nov.** A relatively small collection of isolates from a limited geographic sampling revealed an unexpectedly high level of *Cylindrocladiella* diversity suggesting that many more species in this genus await discovery in South-East Asia.

## Introduction

*Cylindrocladiella* (*Hypocreales*, *Nectriaceae*) are soil-borne fungi that have commonly been confused with the asexual morph of the closely related genus *Calonectria* (Crous 2002). Species of *Cylindrocladiella* can be distinguished from *Calonectria* spp. by their aseptate stipe extensions, distinctive conidiophore branching patterns and their small 1-septate conidia. In addition, they have sexual morphs in *Nectricladiella* that are very different to those in *Calonectria* (Boesewinkel 1982, Crous and Wingfield 1993, Schoch et al. 2000, Crous 2002). Multigene phylogenetic inference has led to the description of a relatively large number of novel species and to the delimitation of cryptic species (Schoch et al. 2000, van Coller et al. 2005, Lombard et al. 2012, 2017). Currently, *Cylindrocladiella* accommodates 35 species (Crous 2002, van Coller et al. 2005, Inderbitzin et al. 2012, Lombard et al. 2012, 2017, Crous et al. 2017).

Species of *Cylindrocladiella* are distributed globally, especially in the tropical, sub-tropical and temperate regions of the world (Crous 2002, Lombard et al. 2012). These fungi are not typically considered primary pathogens although their role in causing plant disease is likely underestimated. The fact that they are isolated using baiting with living plant tissue similar to the approach for *Calonectria* spp. (Crous 2002), suggests some level of pathogenicity. Disease symptoms that have been associated with *Cylindrocladiella* include leaf spot (Mohanan and Sharma 1985, Crous et al. 1991, Crous and Wingfield 1993), damping off (Sharma and Mohanan 1982, Scattolin and Montecchio 2007) and shoot die-back (Brielmaier-Liebetanz et al. 2013). *Cylindrocladiella* spp. are, however, most frequently associated with root diseases (Crous et al. 1991, Crous and Wingfield 1993, Crous 2002). They have, for example, been reported causing root rot on *Eucalyptus* spp. (Mohanan and Sharma 1985, Crous and Wingfield 1993) and *Pinus* sp. (Boesewinkel 1982) in forestry nurseries. They have also been associated with root rot of peanut (Crous and Wingfield 1993), tea (Peerally 1974), kiwi fruit (Erper et al. 2013) and black-foot disease of grapevines (Agustí-Brisach and Armengol 2013, Armengol and Gramaje 2016, Carlucci et al. 2017).

Thirteen species of *Cylindrocladiella* have been reported from South-East Asia from Indonesia and Thailand (Crous 2002, Lombard et al. 2012, 2017). Of these, only four species (*Cy. camelliae*, *Cy. infestans*, *Cy. microcylindrica* and *Cy. viticola*), have been isolated from plant tissues, with the other nine species having been isolated from soil (Crous 2002, Lombard et al. 2012, 2017). However, nothing is known regarding their role as plant pathogens in this region.

In order to provide a better understanding about the diversity of *Cylindrocladiella* species in South-East Asia, this study aimed at identifying a collection of *Cylindrocladiella* isolates obtained from soils collected in plantations and nurseries in Malaysia and Vietnam. This was achieved using multigene sequence comparisons and morphological observations.

## Materials and methods

### Isolates

Soil samples were collected from various plantations and nurseries in Malaysia and Vietnam and baited with germinating alfalfa (*Medicago sativa*) seeds as described by Crous (2002). Direct isolations from fungal structures were made on to malt extract agar (MEA; 2 % w/v; Biolab, Midrand, South Africa). Cultures were incubated for 3–7 d at 25 °C and purified by transferring single hyphal tips from primary isolations to fresh MEA plates. Cultures were deposited in the culture collection (CMW) of the Forestry and Agricultural Biotechnology Institute (FABI), University of Pretoria, South Africa with representative isolates in the collection of the Westerdijk Fungal Biodiversity Institute (CBS), Utrecht, The Netherlands. Dried specimens were deposited in the National Collection of Fungi (PREM), Pretoria, South Africa.

### DNA sequencing and phylogenetic analyses

Seven-day-old fungal cultures grown on MEA at 25 °C were used for DNA extraction using Prepman® Ultra Sample Preparation Reagent (Thermo Fisher Scientific, Waltham, MA, USA) following the protocols provided by the manufacturer. Four loci were amplified and sequenced including the internal transcribed spacer (ITS) region using primers ITS1F (Gardes and Bruns 1993) and ITS4 (White et al. 1990); partial fragments of the translation elongation factor 1-alpha (*tef1*) gene region using primers EF1-728F (Carbone and Kohn 1999) and EF-2 (O’Donnell et al. 1998); partial fragments of the β-tubulin (*tub2*) gene region using primers T1 (O’Donnell and Cigelnik 1997) and CYLTUB1R (Crous et al. 2004a) and part of the Histone H3 (*his3*) gene region using primers CYLH3F and CYLH3R (Crous et al. 2004a).

The PCR reactions were conducted as described by Pham (2018). Amplified fragments were purified using ExoSAP-IT PCR Product Cleanup Reagent (Thermo Fisher Scientific, Waltham, MA, USA). The products were sequenced in both directions with the same primers used for amplification, using the BigDye terminator sequencing kit v. 3.1 (Applied Biosystems, USA) on an ABI PRISM 3100 DNA sequencer (Applied Biosystems, USA).

Raw sequences were assembled and edited using Geneious v. 7.0 (Kearse et al. 2012). Sequence data were compared with other closely related *Cylindrocladiella* spp. available on the GenBank database. Sequences were aligned using MAFFT v. 7 (Katoh and Standley 2013), then edited manually in MEGA v. 7 (Kumar et al. 2016).

Maximum Parsimony (MP) and Maximum Likelihood (ML) analyses were performed on data sets for each gene region and the combined data set. For MP, analyses were conducted using PAUP v. 4.0b10 (Swofford 2003) with phylogenic relationships estimated by heuristic searches with 1000 random stepwise addition sequences and tree bisection and reconstruction (TBR) branch-swapping. Alignment gaps were treated as missing data and all characters were weighted equally. Measures calculated for parsimony included tree length (TL), retention index (RI), consistency index (CI), rescaled consistency index (RC) and homoplasy index (HI). Statistical support for branch nodes in the most parsimonious trees was obtained by performing 1000 bootstrap replicates. For ML, the appropriate substitution model was obtained using the software package jModeltest v. 2.1.5 (Posada 2008). The ML phylogenetic trees were generated using PhyML v. 3.0 (Guindon and Gascuel 2003). Confidence levels for the nodes were determined using 1000 replication bootstrap analyses. For both MP and ML, *Calonectria brachiatica* (CMW 25307) and *Calonectria pauciramosa* (CMW 5638) were used as the outgroup taxa. All resulting trees were viewed using MEGA v. 7 (Kumar et al. 2016).

### Taxonomy

Morphological characteristics were assessed using single hyphal tip cultures on synthetic low-nutrient agar (SNA; Nirenburg 1981) and incubated at 25 °C for 3–7 d. In some cases, pieces of carnation leaf were added to the media to induce sporulation. Fungal structures were studied by mounting in 80 % lactic acid on glass sides and examined using a Nikon H550L microscope (Nikon, Japan). Thirty to fifty measurements were made for all taxonomically informative characters depending on their availability. The 95 % confidence levels were determined and extremes of conidial measurements are given in parentheses. For all other fungal structures, only extremes are presented. Colony colour and morphology were assessed using 7-d-old cultures on MEA grown at 25 °C using the colour charts of Rayner (1970). To determine the optimal temperature for growth, cultures were transferred to MEA and incubated at temperatures ranging from 5 to 35 °C at 5 °C intervals. Fungal descriptions and associated metadata were deposited in MycoBank (Crous et al. 2004b).

## Results

### Isolates

Nineteen isolates in total were obtained from soil baits. Of these, 15 were from Vietnam (nine from Tuyen Quang, four from Nghe An, one from Vinh Phuc and one from Hanoi) and four were from Sabah, Malaysia. The majority (16) of the isolates were from soils collected from *Acacia* plantations (Table 1).

**Table 1.** Collection details and GenBank accessions of *Cylindrocladiella* isolates included in the phylogenetic analysis.

Species	Isolate number^1,3^	Substrate	Locality	Genbank accession^2^				References
				*tub2*	*his3*	*tef1*	ITS	
*Cy. arbusta*	**CMW 47295^T^; CBS 143546**	soil in *Acacia mangium* plantation	Tan Ky, Nghe An, Vietnam	MH016958	MH016996	MH016977	MH017015	This study
	**CMW 47296; CBS 143547**	soil in *A. mangium* plantation	Tan Ky, Nghe An, Vietnam	MH016959	MH016997	MH016978	MH017016	This study
*Cy. camelliae*	CPC 234; PPRI 3990; IMI 346845	*Eucalyptus grandis*	South Africa	AY793471	AY793509	JN099087	AF220952	Boesewinkel 1982
	CPC 237	*E. grandis*	South Africa	JN098749	JN098839	JN099090	JN100573	Boesewinkel 1982
*Cy. clavata*	CBS 129563; CPC 17591	soil	Australia	JN098751	JN098859	JN098975	JN099096	Lombard et al. 2012
	CBS 129564^T^; CPC 17592	soil	Australia	JN098752	JN098858	JN098974	JN099095	Lombard et al. 2012
*Cy. cymbiformis*	CBS 129553^T^; CPC 17393	soil	Australia	JN098753	JN098866	JN098988	JN099103	Lombard et al. 2012
*Cy. elegans*	CBS 338.92^T^; PPRI 4050; IMI 346847	leaf litter	South Africa	AY793474	AY793512	JN099039	AY793444	Crous and Wingfield 1993
	CBS 110801; CPC 525	leaf litter	South Africa	JN098755	JN098916	JN099044	JN100609	Crous and Wingfield 1993
*Cy. lageniformis*	CBS 340.92^T^; PPRI 4449; UFV 115	*Eucalyptus* sp.	Brazil	AY793481	AY793520	JN099003	AF220959	Crous and Wingfield 1993
	CBS 111060; CPC 1240	*Eucalyptus* sp.	South Africa	JN098770	JN098918	JN099046	JN100611	Crous and Wingfield 1993
	**CMW 47419**	soil in *E. camaldulensis* plantation	Hoang Mai, Nghe An, Vietnam	MH016972	MH017010	MH016991	MH017029	This study
*Cy. lanceolata*	CBS 129565; CPC 17566	soil	Australia	JN098788	JN098939	JN099069	JN100632	Lombard et al. 2012
	CBS 129566^T^; CPC 17567	soil	Australia	JN098789	JN098862	JN098978	JN099099	Lombard et al. 2012
*Cy. longiphialidica*	CBS 129557^T^; CPC 18839	soil	Thailand	JN098790	JN098851	JN098966	JN100585	Lombard et al. 2012
	CBS 129558	soil	Thailand	JN098791	JN098852	JN098967	JN100586	Lombard et al. 2012
*Cy. malesiana*	**CMW 48276; CBS 143549**	soil in *A. mangium* plantation	Tawau, Sabah, Malaysia	MH016960	MH016998	MH016979	MH017017	This study
	**CMW 48277; CBS 143550**	soil in *A. mangium* plantation	Tawau, Sabah, Malaysia	MH016961	MH016999	MH016980	MH017018	This study
	**CMW 48278^T^; CBS 143548**	soil in *A. mangium* plantation	Tawau, Sabah, Malaysia	MH016962	MH017000	MH016981	MH017019	This study
*Cy. malesiana*	**CMW 48279**	soil in *A. mangium* plantation	Tawau, Sabah, Malaysia	MH016963	MH017001	MH016982	MH017020	This study
*Cy. microcylindrica*	CBS 111794^T^; ATCC 38571; CPC 2375	*Echeveria elegans*	Indonesia	AY793483	AY793523	JN099041	AY793452	Schoch et al. 2000
*Cy. natalensis*	CBS 110800; CPC 529	soil	South Africa	JN098793	JN098915	JN099043	JN100608	Lombard et al. 2012
	CBS 114943^T^; CPC 456	*Arachis hypogaea*	South Africa	JN098794	JN098895	JN099016	JN100588	Lombard et al. 2012
*Cy. nederlandica*	CBS 143.95; PD94/1353	*Kalanchoe* sp.	The Netherlands	JN098798	JN098891	JN099013	JN099129	Lombard et al. 2012
	CBS 152.91^T^; PD90/2015	*Pelargonium* sp.	The Netherlands	JN098800	JN098910	JN099033	JN100603	Lombard et al. 2012
*Cy. novaezelandica*	CBS 486.77^T^; ATCC 44815; CPC 2397	*Rhododendron indicum*	New Zealand	AY793485	AY793525	JN099050	AF220963	Boesewinkel 1982
*Cy. obpyriformis*	**CMW 47194^T^; CBS 143552**	soil in *Acacia* hybrid plantation	Tuyen Quang, Vietnam	MH016965	MH017003	MH016984	MH017022	This study
	**CMW 49940; CBS 143553**	soil in *Camellia chrysantha* nursery	Tam Dao, Vinh Phuc, Vietnam	MH016966	MH017004	MH016985	MH017023	This study
*Cy. parvispora*	**CMW 47193**	soil in *Acacia* hybrid plantation	Tuyen Quang, Vietnam	MH016967	MH017005	MH016986	MH017024	This study
	**CMW 47197^T^; CBS 143554**	soil in *Acacia* hybrid plantation	Tuyen Quang, Vietnam	MH016968	MH017006	MH016987	MH017025	This study
	**CMW 47207; CBS 143555**	soil in *Acacia* hybrid plantation	Tuyen Quang, Vietnam	MH016969	MH017007	MH016988	MH017026	This study
	**CMW 47208; CBS 143556**	soil in *Acacia* hybrid plantation	Tuyen Quang, Vietnam	MH016970	MH017008	MH016989	MH017027	This study
	**CMW 47315**	soil in *A. mangium* plantation	Son Duong, Tuyen Quang, Vietnam	MH016971	MH017009	MH016990	MH017028	This study
*Cy. peruviana*	CBS 113022; CPC 4291	*Eucalyptus* sp.	South Africa	JN098801	JN098906	JN099029	JN100599	Boesewinkel 1982
	CPC 2404^T^; IMUR 1843	ants	Peru	AY793500	AY793540	JN098968	AF220966	Boesewinkel 1982
	**CMW 47297**	soil in *A. mangium* plantation	Tan Ky, Nghe An, Vietnam	MH016973	MH017011	MH016992	MH017030	This study
	**CMW 47304**	soil in *A. mangium* plantation	Son Duong, Tuyen Quang, Vietnam	MH016974	MH017012	MH016993	MH017031	This study
	**CMW 47333**	soil in *A. mangium* plantation	Son Duong, Tuyen Quang, Vietnam	MH016975	MH017013	MH016994	MH017032	This study
*Cy. peruviana*	**CMW 47416**	soil	Bac Tu Liem, Hanoi, Vietnam	MH016976	MH017014	MH016995	MH017033	This study
*Cy. pseudocamelliae*	CBS 129555^T^; CPC 18825	soil	Thailand	JN098814	JN098843	JN098958	JN100577	Lombard et al. 2012
	CBS 129556; CPC 18832	soil	Thailand	JN098815	JN098846	JN098961	JN100580	Lombard et al. 2012
*Cy. solicola*	**CMW 47198^T^; CBS 143551**	soil in *Acacia* hybrid plantation	Tuyen Quang, Vietnam	MH016964	MH017002	MH016983	MH017021	This study
*Cy. variabilis*	CBS 375.93; IMI 317057	*Mangifera indica*	India	JN098836	JN098881	JN099000	JN099119	Lombard et al. 2012
	CBS 129561^T^; CPC 17505	soil	Australia	JN098719	JN098950	JN099080	JN100643	Lombard et al. 2012

^1^ CBS: Culture collection of Westerdijk Fungal Biodiversity Institute (WI), Utrecht, the Netherlands; CMW: Culture collection of the Forestry and Agricultural Biotechnology Institute (FABI), University of Pretoria, Pretoria, South Africa; CPC: Pedro Crous working collection housed at WI; IMI: International Mycological Institute, CABI-Bioscience, Egham, Bakeham Lane, UK; IMUR: Institute of Mycology, University of Recife, Recife, Brazil; ATCC: American Type Culture Collection, Virginia, U.S.A; PPRI: Plant Protection Research Institute, Agricultural Research Council, Pretoria, South Africa; UFV: Universidade Federal de Viçosa, Viçosa, Brazil.

^2^*tub2* = β-tubulin;

*his3* = histone H3;

*tef1* = translation elongation factor 1-alpha; ITS = Internal transcribed spacer regions 1 and 2 and the 5.8S gene of the ribosomal RNA.

^T^ Ex-type cultures.

^3^ Isolates obtained during the survey in this study are indicated in **bold.**

### Phylogenetic analyses

Approximately 500–570 bases were obtained for each of the *his3, tef1, tub2* and ITS loci. For the ML analyses of each individual data sets, the TIM2+G model was selected for *his3*; GTR+G model for *tef1*; TrN+I+G for *tub2* and the K80+I+G for ITS. The ML tree of each individual gene region with bootstrap support values of both the ML and MP analyses are presented in Suppl. materials 1–4.

**Figure 1.**
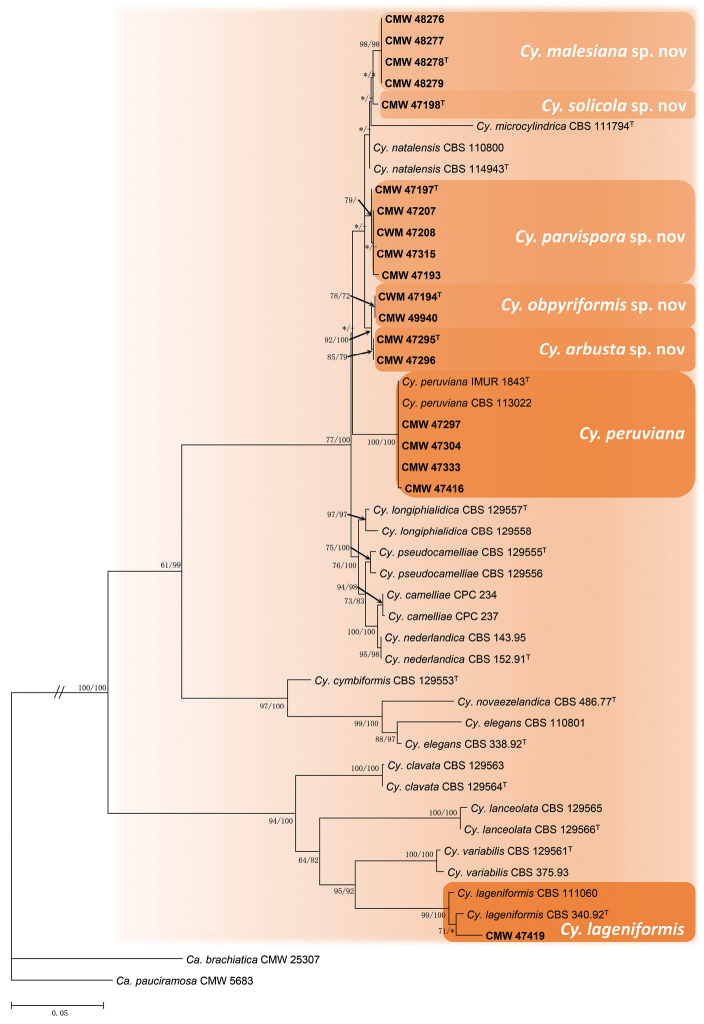
Phylogenetic tree based on maximum likelihood (ML) analysis of a combined data set of *his3, tef1, tub2* and ITS sequence alignments. Bootstrap value ≥ 60 % for maximum parsimony (MP) and ML analyses are indicated at the nodes. Bootstrap values lower than 60 % are marked with “*” and absent are marked with “–”. Isolates representing ex–type material are marked with “T” and isolates collected in this study are highlighted in **bold**. *Calonectria brachiatica* (CMW 25307) and *Calonectria pauciramosa* (CMW 5683) represent the outgroups.

The combined data set of *his3, tef1, tub2* and ITS, included 44 ingroup taxa and two outgroup taxa. The data set consisted of 2054 characters, of which 640 were parsimony-informative and 1414 characters were excluded. The MP analysis yielded 1000 trees (TL = 1414; CI = 0.691; RI = 0.880; RC = 0.608; HI = 0.309). The TIM2+I+G model was selected for the combined data set for the ML analyses. The ML tree with bootstrap support values of both the ML and MP analyses is presented in Figure 1.

In the phylogenetic tree (Figure 1), four isolates (CMW 47297, CMW 47304, CMW 47333, CMW 47416) clustered in the clade representing *Cy. peruviana* (ex-type IMUR 1843). *Cylindrocladiella lageniformis* (ex-type CBS 340.92) was represented by CMW 47419. The remaining isolates resided in five distinct clades representing novel taxa, accommodating four isolates (CMW 48276, CMW 48277, CMW 48278, CMW 48279), one isolate (CMW 47198), five isolates (CMW 47193, CMW 47197, CMW 47207, CMW 47208, CMW 47315), two isolates (CMW 47194, CMW 49940) and two isolates (CMW 47295, CMW 47296) respectively.

### Taxonomy

Morphological comparisons and phylogenetic inference showed that 19 *Cylindrocladiella* isolates represented five novel species along with two previously described species, *Cy. lageniformis* (CMW 47419) and *Cy. peruviana* (CMW 47297, CMW 47304, CMW 47333, CMW 47416). The novel taxa are provided with names in *Cylindrocladiella* and their important morphological characteristics are compared in Table 2.

**Table 2.** Comparisons of morphological characteristics of *Cylindrocladiella* spp. included in this study.

Species	Stipe extension	Vesicle		Macroconidia		Subverticillate conidiophores	References
	Length (µm)	Diam (µm)	Shape	Size (µm)	Average (µm)		
***Cy. arbusta***	93–139	4–5.5	obpyriform to lanceolate	(8.5–)10–12 (–13.5) × 2–3	11 × 2.5	moderate	This study
***Cy. malesiana***	114.5–144.5	4.5–6	fusoid to lanceolate	(10–)11–13(–13.5) × (1.5–)2–2.5	12 × 2	abundant	This study
***Cy. microcylindrica***	70–130	3–4	cylindrical to lanceolate	(10–)12–14 (–15) × 2(–3)	12.5 × 2	abundant	Schoch et al. 2000
***Cy. natalensis***	82–127	6–8	ellipsoidal to fusoid	(12–)14–16 (–17) × 2–3	15× 3	moderate	Lombard et al. 2012
***Cy. obpyriformis***	86.5–150	4–7	obpyriform	(9–)11–13(–15) × 2–3(–3.5)	12 × 2.5	abundant	This study
***Cy. parvispora***	112.5–141	4.5–6.5	fusoid to cylindrical	(8–)10– 12 (–13) × 2–2.5	11 × 2	moderate	This study
***Cy. solicola***	93.5–170	3.5–6.5	broadly clavate to lanceolate to fusiform	(10.5–)12.5–14.5(–15.5) × 2–3	13.5 × 2.5	abundant	This study

#### Cylindrocladiella arbusta

Taxon classificationFungiHypocrealesNectriaceae

N.Q. Pham, T.Q. Pham & M.J. Wingf. sp. nov.

MB824550

Figure 2

##### Etymology.

Name refers to a plantation and the environment where this fungus was isolated.

##### Type material.

VIETNAM. Nghe An Province: Tan Ky, from soil in *Acacia mangium* plantation, Nov. 2013, N.Q. Pham & T.Q. Pham, herbarium specimen of dried culture, PREM 62159 (holotype), CMW 47295 = CBS 143546 (ex-type culture).

**Figure 2.**
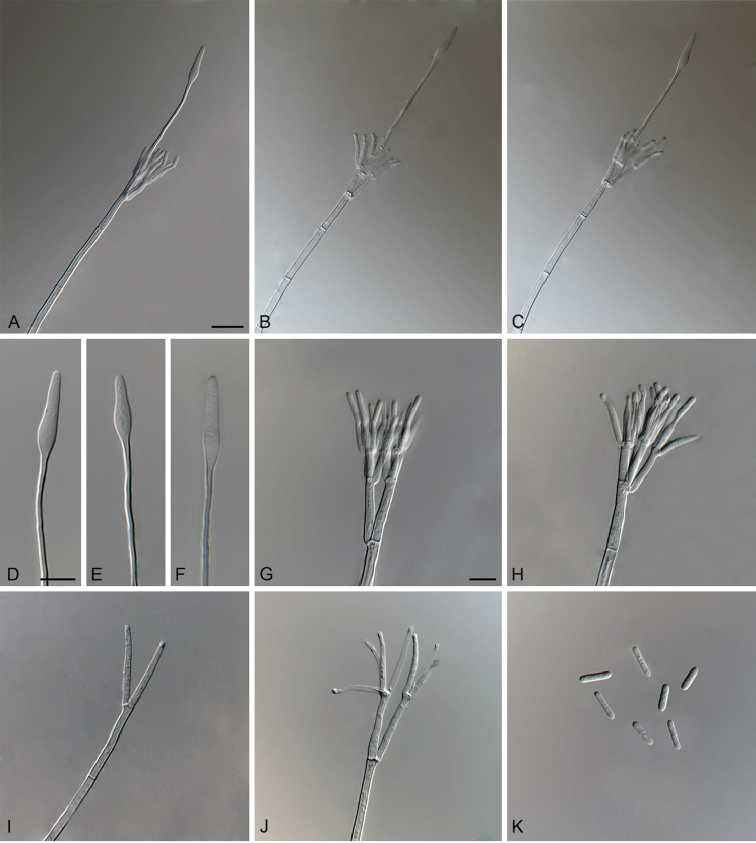
*Cylindrocladiella arbusta* (ex-type CMW 47295). **A–C** Penicillate conidiophores **D–F** Obpyriform to lanceolate vesicles **G–H** Penicillate conidiogenous apparatus **I–J** Subverticillate conidiophores **K** Conidia. Scale bars: **A** = 20 µm (apply to **B–C**); **D** = 10 µm (apply to **E–F**); **G** = 10 µm (apply to **H–K**).

##### Description.

*Sexual morph* not observed. *Conidiophores* dimorphic, penicillate and subverticillate, mononematous and hyaline. *Penicillate conidiophores* comprising a stipe, a penicillate arrangement of fertile branches, a stipe extension and a terminal vesicle; stipe septate, hyaline, smooth, 116–166.5 × 4–5 µm; stipe extension aseptate, straight, 93–139 µm long, thick-walled with one basal septum, terminating in thin-walled, obpyriform to lanceolate vesicles, 4–5.5 µm wide. *Penicillate conidiogenous apparatus* with primary branches aseptate, 15–28.5 × 2.5–5 µm, secondary branches aseptate, 12–22.5 × 2.5–3.5 µm, each terminal branch producing 2–4 phialides; phialides doliiform to reniform to cymbiform, hyaline, aseptate, 10–18 × 2–3 µm, apex with minute periclinal thickening and collarette. *Subverticillate conidiophores* in moderate numbers, comprising of a septate stipe and primary branches terminating in 2–4 phialides; primary branches straight, hyaline, 0–1-septate, 25–31 × 2.5–3.5 µm; phialides cymbiform to cylindrical, hyaline, aseptate, 16.5–30.5 × 2–3.5 µm, apex with minute periclinal thickening and collarette. *Conidia* cylindrical, rounded at both ends, straight, 1-septate, (8.5–)10–12(–13.5) × 2–3 µm (av. = 11 × 2.5 µm), frequently slightly flattened at the base, held in asymmetrical clusters by colourless slime.

##### Culture characteristics.

Colonies white to buff on the surface and salmon to sienna in reverse on MEA after 7 d; smooth margins; extensive aerial mycelium in the middle and the margins; chlamydospores moderate, arranged in chains. Optimal growth temperature at 25 °C, no growth at 5 °C and 35 °C; after 7 d, colonies at 10 °C, 15 °C, 20 °C, 25 °C and 30 °C reached 3.5 mm, 27.7 mm, 49.2 mm, 67.9 mm and 52.7 mm, respectively.

##### Additional material examined.

VIETNAM, Nghe An Province: Tan Ky, from soil in *Acacia mangium* nursery, Nov. 2013, N.Q. Pham & T.Q. Pham, PREM 62160, culture CMW 47296 = CBS 143547.

##### Distribution.

Nghe An, Vietnam.

##### Notes.

*Cylindrocladiella arbusta* is phylogenetically closely related to *Cy. natalensis*, *Cy. obpyriformis* and *Cy. parvispora*. The stipe extensions of *Cy. arbusta* are longer than those of *Cy. natalensis* and shorter than those of *Cy. obpyriformis* and *Cy. parvispora*. Conidia of *Cy. arbusta* are shorter than those of *Cy. natalensis* and *Cy. obpyriformis* (Table 2).

#### Cylindrocladiella malesiana

Taxon classificationFungiHypocrealesNectriaceae

N.Q. Pham & M.J. Wingf. sp. nov.

MB824551

Figure 3

##### Etymology.

Name refers to Malaysia, the country where this species was first collected.

##### Type material.

MALAYSIA. Sabah State: Tawau, Brumas, from soil in *Acacia mangium* plantation, Mar. 2013, M.J. Wingfield, herbarium specimen of dried culture, PREM 62161 (holotype), CMW 48278 = CBS 143548 (ex-type culture).

**Figure 3.**
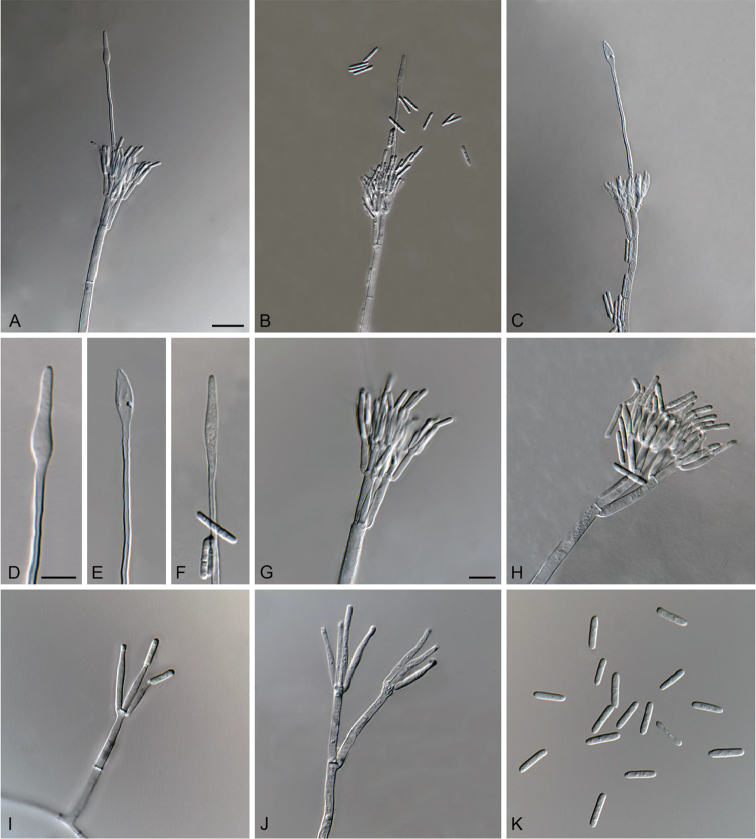
*Cylindrocladiella malesiana* (ex-type CMW 48278). **A–C** Penicillate conidiophores **D–F** Fusoid to lanceolate vesicles **G–H** Penicillate conidiogenous apparatus **I–J** Subverticillate conidiophores **K** Conidia. Scale bars: **A** = 20 µm (apply to **B–C**); **D** = 10 µm (apply to **E–F**); **G** = 10 µm (apply to **H–K**).

##### Description.

*Sexual morph* not observed. *Conidiophores* dimorphic, penicillate and subverticillate, mononematous and hyaline. *Penicillate conidiophores* comprising a stipe, a penicillate arrangement of fertile branches, a stipe extension and a terminal vesicle; stipe septate, hyaline, smooth, 76.5–126 × 3.5–5 µm; stipe extension aseptate, straight, 114.5–144.5 µm long, thick-walled with one basal septum, terminating in thin-walled, fusoid to lanceolate vesicles, 4.5–6 µm wide. *Penicillate conidiogenous apparatus* with primary branches aseptate, 16.5–24 × 3–4.5 µm, secondary branches aseptate, 10.5–15 × 2–3.5 µm, each terminal branch producing 2–4 phialides; phialides cymbiform to cylindrical, hyaline, aseptate, 9–15.5 × 2–3.5 µm, apex with minute periclinal thickening and collarette. *Subverticillate conidiophores* abundant, comprising of a septate stipe and primary branches terminating in 2–4 phialides; primary branches straight, hyaline, 0–1-septate, 13.5–35 × 2.5–4 µm; phialides cymbiform to cylindrical, hyaline, aseptate, 14.5–27 × 2–3.5 µm, apex with minute periclinal thickening and collarette. *Conidia* cylindrical, rounded at both ends, straight, 1-septate, (10–)11–13(–13.5) × (1.5–)2–2.5 µm (av. = 12 × 2 µm), frequently slightly flattened at the base, held in asymmetrical clusters by colourless slime.

##### Culture characteristics.

Colonies buff to hazel on the surface and dark brick to brown vinaceous in reverse on MEA after 7 d; smooth to undulate margins; moderate aerial mycelium; chlamydospores moderate, arranged in chains. Optimal growth temperature at 25 °C, no growth at 5 °C and 35 °C; after 7 d, colonies at 10 °C, 15 °C, 20 °C, 25 °C and 30 °C reached 3.8 mm, 24.3 mm, 45.2 mm, 74.4 mm and 48.8 mm, respectively.

##### Distribution.

Sabah, Malaysia

##### Additional material examined.

MALAYSIA. Sabah state: Tawau, Brumas, from soil in *Acacia mangium* plantation, Mar. 2013, M.J. Wingfield, PREM 62162, culture CMW 48276 = CBS 143549; *ibid*., PREM 62163, culture CMW 48277 = CBS 143550.

##### Notes.

*Cylindrocladiella malesiana* is phylogenetically closely related to *Cy. microcylindrica*, *Cy. natalensis* and *Cy. solicola*. Conidia of *Cy. malesiana* are shorter than those of *Cy. microcylindrica*, *Cy. natalensis* and *Cy. solicola* (Table 2).

#### Cylindrocladiella obpyriformis

Taxon classificationFungiHypocrealesNectriaceae

N.Q. Pham, T.Q. Pham & M.J. Wingf. sp. nov.

MB824552

Figure 4

##### Etymology.

Name refers to the obpyriform terminating vesicles in this species.

##### Type material.

VIETNAM. Tuyen Quang Province, from soil in *Acacia* hybrid plantation, Nov. 2013, N.Q. Pham & T.Q. Pham, herbarium specimen of dried culture, PREM 62165 (holotype), CMW 47194 = CBS 143552 (ex-type culture).

**Figure 4.**
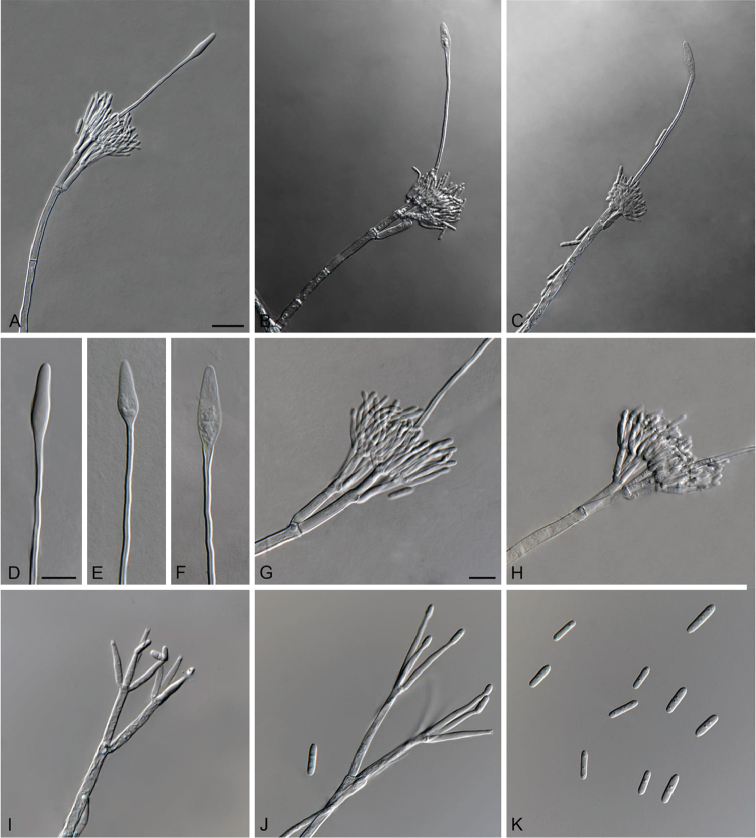
*Cylindrocladiella obpyriformis* (ex-type CMW 47194). **A–C** Penicillate conidiophores **D–F** Obpyriform vesicles **G–H** Penicillate conidiogenous apparatus **I–J** Subverticillate conidiophores **K** Conidia. Scale bars: **A** = 20 µm (apply to **B–C**); **D** = 10 µm (apply to **E–F**); **G** = 10 µm (apply to **H–K**).

##### Description.

*Sexual morph* not observed. *Conidiophores* dimorphic, penicillate and subverticillate, mononematous and hyaline. *Penicillate conidiophores* comprising a stipe, a penicillate arrangement of fertile branches, a stipe extension and a terminal vesicle; stipe septate, hyaline, smooth, 58.5–148 × 4–6 µm; stipe extension aseptate, straight, 86.5–150 µm long, thick-walled with one basal septum, terminating in thin-walled, obpyriform vesicles, 4–7 µm wide. *Penicillate conidiogenous apparatus* with primary branches aseptate, 17.5–31.5 × 3–5 µm, secondary branches aseptate, 10–19 × 2–4 µm, each terminal branch producing 2–4 phialides; phialides cymbiform to cylindrical, hyaline, aseptate, 10.5–18 × 2–3 µm, apex with minute periclinal thickening and collarette. *Subverticillate conidiophores* abundant, comprising of a septate stipe and primary branches terminating in 2–4 phialides; primary branches straight, hyaline, 0–1-septate, 15–38.5 × 2–4 µm; phialides cymbiform to cylindrical, hyaline, aseptate, 13–30.5 × 2–3 µm, apex with minute periclinal thickening and collarette. *Conidia* cylindrical, rounded at both ends, straight, 1-septate, (9–)11–13(–15) × 2–3(–3.5) µm (av. = 12 × 2.5 µm), frequently slightly flattened at the base, held in asymmetrical clusters by colourless slime.

##### Culture characteristics.

Colonies buff to isabelline on the surface and dark brick to sepia in reverse on MEA after 7 d; smooth to undulate margins; extensive aerial mycelium especially in the middle; chlamydospores moderate, arranged in chains. Optimal growth temperature at 25 °C, no growth at 5 °C and 35 °C; after 7 d, colonies at 10 °C, 15 °C, 20 °C, 25 °C and 30 °C reached 5.4 mm, 25.5 mm, 47.2 mm, 74.0 mm and 50.8 mm, respectively.

##### Distribution.

Tuyen Quang & Vinh Phuc, Vietnam

##### Additional material examined.

VIETNAM. Vinh Phuc Province: Tam Dao, from soil in *Camellia chrysantha* nursery, Sept. 2013, N.Q. Pham, Q.N. Dang & T.Q. Pham, PREM 62166, culture CMW 49940 = CBS 143553.

##### Notes.

*Cylindrocladiella obpyriformis* is phylogenetically closely related to *Cy. arbusta*, *Cy. natalensis* and *Cy. parvispora*. The stipe extensions of *Cy. obpyriformis* are longer than those of *Cy. arbusta*, *Cy. natalensis* and *Cy. parvispora* (Table 2).

#### Cylindrocladiella parvispora

Taxon classificationFungiHypocrealesNectriaceae

N.Q. Pham, T.Q. Pham & M.J. Wingfield sp. nov.

MB824553

Figure 5

##### Etymology.

Name refers to the small conidia produced by this species.

##### Type material.

VIETNAM. Tuyen Quang Province, from soil in *Acacia* hybrid plantation, Nov. 2013, N.Q. Pham & T.Q. Pham, herbarium specimen of dried culture, PREM 62167 (holotype), CMW 47197 = CBS 143554 (ex-type culture).

**Figure 5.**
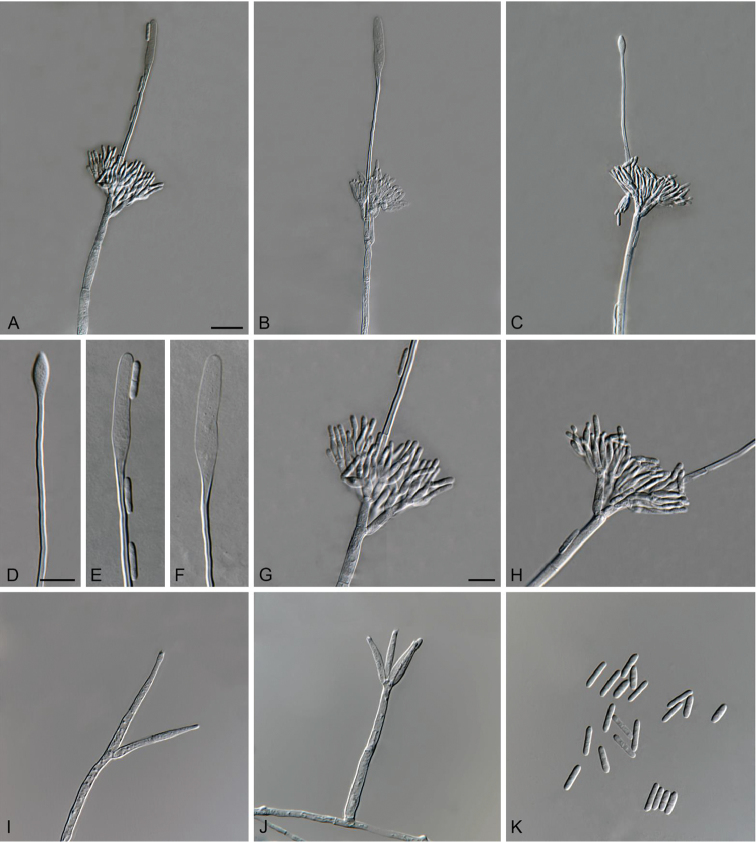
*Cylindrocladiella parvispora* (ex-type CMW 47197). **A–C** Penicillate conidiophores **D–F** Fusoid to cylindrical vesicles **G–H** Penicillate conidiogenous apparatus **I–J** Subverticillate conidiophores **K** Conidia. Scale bars: **A** = 20 µm (apply to **B–C**); **D** = 10 µm (apply to **E–F**); **G** = 10 µm (apply to **H–K**).

##### Description.

*Sexual morph* not observed. *Conidiophores* dimorphic, penicillate and subverticillate, mononematous and hyaline. *Penicillate conidiophores* comprising a stipe, a penicillate arrangement of fertile branches, a stipe extension and a terminal vesicle; stipe septate, hyaline, smooth, 67–107 × 3–6.5 µm; stipe extension aseptate, straight, 112.5–141 µm long, thick-walled with one basal septum, terminating in thin-walled, fusoid to cylindrical vesicles, 4.5–6.5 µm wide. *Penicillate conidiogenous apparatus* with primary branches aseptate, 10.5–25 × 2–4 µm, secondary branches aseptate, 7.5–17 × 2–3 µm, each terminal branch producing 2–4 phialides; phialides doliiform to reniform to cymbiform, hyaline, aseptate, 7.5–13 × 2–3 µm, apex with minute periclinal thickening and collarette. *Subverticillate conidiophores* in moderate numbers, comprising of a septate stipe and primary branches terminating in 2–4 phialides; primary branches straight, hyaline, 0–1-septate, 15.5–27 × 2.5–4 µm; phialides cymbiform to cylindrical, hyaline, aseptate, 13.5–41 × 2.5–6 µm, apex with minute periclinal thickening and collarette. *Conidia* cylindrical, rounded at both ends, straight, 1-septate, (8–)10– 12(–13) × 2–2.5 µm (av. = 11 × 2 µm), frequently slightly flattened at the base, held in asymmetrical clusters by colourless slime.

##### Culture characteristics.

Colonies buff to honey to isabelline on the surface and umber to sepia in reverse on MEA after 7 d; smooth to undulate margin; abundant aerial mycelium especially in the middle; chlamydospores moderate, arranged in chains. Optimal growth temperature at 25 °C, no growth at 5 °C and 35 °C; after 7 d, colonies at 10 °C, 15 °C, 20 °C, 25 °C and 30°C reached 5.5 mm, 23.4 mm, 43.8 mm, 63.6 mm and 49.2 mm, respectively.

##### Distribution.

Tuyen Quang, Vietnam

##### Additional material examined.

VIETNAM. Tuyen Quang Province, from soil in *Acacia* hybrid plantation, Nov. 2013, N.Q. Pham & T.Q. Pham, PREM 62168, culture CMW 47207 = CBS 143555; *ibid*., PREM 62169, culture CMW 47208 = CBS 143556.

##### Notes.

*Cylindrocladiella parvispora* is phylogenetically closely related to *Cy. arbusta*, *Cy. natalensis* and *Cy. obpyriformis*. Conidia of *Cy. parvispora* are slightly smaller than those of *Cy. arbusta*, *Cy. natalensis* and *Cy. obpyriformis* (Table 2).

#### Cylindrocladiella solicola

Taxon classificationFungiHypocrealesNectriaceae

N.Q. Pham, T.Q. Pham & M.J. Wingf. sp. nov.

MB824554

Figure 6

##### Etymology.

Name refers to soil, the substrate from which this fungus was first isolated.

##### Type material.

VIETNAM. Tuyen Quang Province, from soil in *Acacia* hybrid plantation, Nov. 2013, N.Q. Pham & T.Q. Pham, herbarium specimen of dried culture, PREM 62164 (holotype), CMW 47198 = CBS 143551 (ex-type culture).

**Figure 6.**
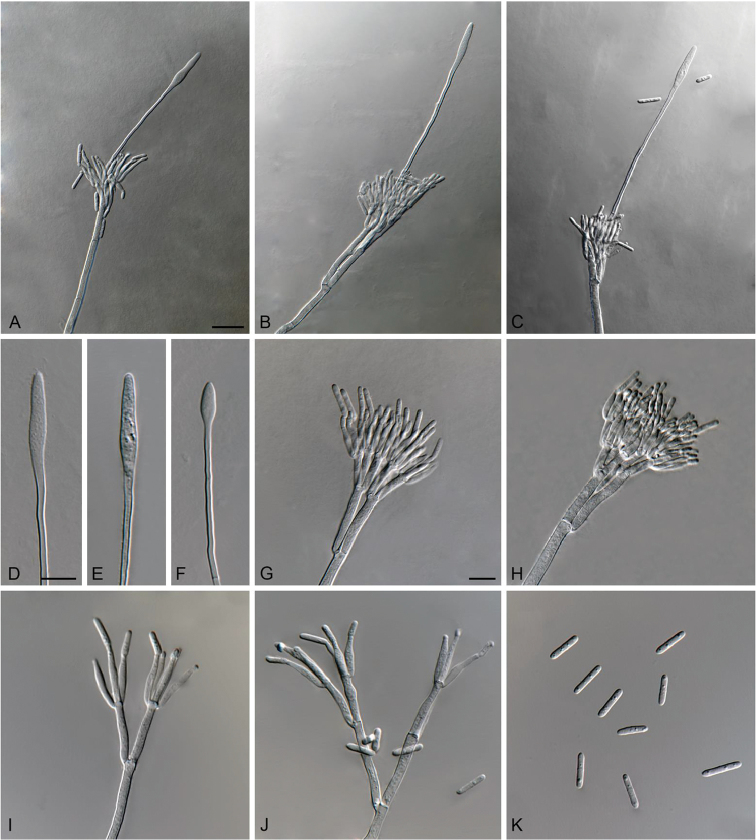
*Cylindrocladiella solicola* (ex-type CMW 47198). **A–C** Penicillate conidiophores **D–F** Broadly clavate to lanceolate to fusiform vesicles **G–H** Penicillate conidiogenous apparatus **I–J** Subverticillate conidiophores **K** Conidia. Scale bars: **A** = 20 µm (apply to **B–C**); **D** = 10 µm (apply to **E–F**); **G** = 10 µm (apply to **H–K**).

##### Description.

*Sexual morph* not observed. *Conidiophores* dimorphic, penicillate and subverticillate, mononematous and hyaline. *Penicillate conidiophores* comprising a stipe, a penicillate arrangement of fertile branches, a stipe extension and a terminal vesicle; stipe septate, hyaline, smooth, 58.5–120 × 2.5–5 µm; stipe extension aseptate, straight, 93.5–170 µm long, thick-walled with one basal septum, terminating in thin-walled, broadly clavate to lanceolate to fusiform vesicles, 3.5–6.5 µm wide. *Penicillate conidiogenous apparatus* with primary branches aseptate, 16–36.5 × 3–4.5 µm, secondary branches aseptate, 10–16 × 2.5–3.5 µm, each terminal branch producing 2–4 phialides; phialides cymbiform to cylindrical, hyaline, aseptate, 9–15.5 × 2–3 µm, apex with minute periclinal thickening and collarette. *Subverticillate conidiophores* abundant, comprising of a septate stipe and primary branches terminating in 2–4 phialides; primary branches straight, hyaline, 0–1-septate, 16.5–25 × 2.5–5 µm; phialides cymbiform to cylindrical, hyaline, aseptate, 12–28 × 2.5–4 µm, apex with minute periclinal thickening and collarette. *Conidia* cylindrical, rounded at both ends, straight, 1-septate, (10.5–)12.5–14.5(–15.5) × 2–2.5(–3) µm (av. = 13.5 × 2.5 µm), frequently slightly flattened at the base, held in asymmetrical clusters by colourless slime.

##### Culture characteristics.

Colonies honey to isabelline on the surface and sepia to brown vinaceous in reverse on MEA after 7 d; undulate margins; extensive aerial mycelium especially in the middle; chlamydospores moderate, arranged in chains. Optimal growth temperature at 25 °C, no growth at 5 °C and 35 °C; after 7 d, colonies at 10 °C, 15 °C, 20 °C, 25 °C and 30 °C reached 5.2 mm, 20.4 mm, 37.8 mm, 61.2 mm and 37.1 mm, respectively.

##### Distribution.

Tuyen Quang, Vietnam

##### Notes.

*Cylindrocladiella solicola* is phylogenetically closely related to *Cy. malesiana*, *Cy. microcylindrica* and *Cy. natalensis*. The stipe extensions of *Cy. solicola* are longer than those of *Cy. malesiana*, *Cy. microcylindrica* and *Cy. natalensis* (Table 2).

## Discussion

Application of multigene phylogenetic inference made it possible to identify five novel and two known species of *Cylindrocladiella* in this study. The seven species found bring the number of *Cylindrocladiella* known from South-East Asia to 20 (Crous 2002, Lombard et al. 2012, 2017), thus suggesting that this geographical region could be a possible centre of diversity for the genus *Cylindrocladiella*. A relatively small collection of isolates was shown to represent a high diversity of *Cylindrocladiella* spp. This indicates that more *Cylindrocladiella* spp. remain to be discovered in South-East Asia.

The *his3* gene region provided the best resolution for species delineation amongst the four gene regions applied. This was the only gene region that could distinguish between all five novel species in the study. The ITS could not resolve any single lineage and the *tef1* gene region failed to distinguish between *Cy. arbusta* and *Cy. parvispora*. The phylogenetic relationship between *Cy. arbusta*, *Cy. malesiana* and *Cy. obpyriformis* could not be resolved using the *tub2* gene region (Suppl. materials 1–4). In the most recent study of species of *Cylindrocladiella* (Lombard et al. 2017), the *his3* gene region was not used in the analyses because it provided limited information compared with *tef1* and *tub2* gene sequences that were more informative. However, the results of the present study suggest that *his3* sequence data should be included in future studies as they provide valuable additional information on the relationships amongst some groups of species.

Five novel species, described as *Cy. arbusta*, *Cy. malesiana*, *Cy. obpyriformis, Cy. parvispora* and *Cy. solicola*, were all isolated from soil samples associated with *Acacia* plantations across Malaysia and Vietnam. In comparison with a previous study on *Calonectria* spp. from South-East Asia (Pham 2018), even though they share similar ecological niches, *Cylindrocladiella* spp. seemed to have a relatively narrow distribution and host association. This suggests that there is some substrate specialisation for these species of *Cylindrocladiella*. It is possible that they are mild pathogens of roots but no evidence of disease was observed.

This study includes the first report of *Cy. lageniformis* and *Cy. peruviana* in Vietnam. These two species have been reported as causal agents of black-foot disease, one of the most economically important fungal disease and a major constraint to wine and grape production (van Coller et al. 2005, Koike et al. 2016). The detection of these species from plantations soils in Vietnam might suggest that they infect the roots of *Acacia* spp. but this would require further investigation. These species have also been reported to cause leaf spots as well as root and cutting rot of *Eucalyptus* in Brazil (Crous et al. 1991, Crous and Wingfield 1993) and they clearly deserve further study in South-East Asia.

## Supplementary Material

XML Treatment for Cylindrocladiella arbusta

XML Treatment for Cylindrocladiella malesiana

XML Treatment for Cylindrocladiella obpyriformis

XML Treatment for Cylindrocladiella parvispora

XML Treatment for Cylindrocladiella solicola

## Supplementary materials

Supplementary material 1 Figure S1. Phylogenetic tree based on maximum likelihood (ML) analysis of *his*3 sequence alignments Data type: molecular data Explanation note: Bootstrap value ≥ 60 % for maximum parsimony (MP) and ML analyses are indicated at the nodes. Bootstrap values lower than 60 % are marked with “*” and absent are marked with “–”. Isolates representing ex–type material are marked with “T” and isolates collected in this study are highlighted in **bold**. *Calonectria brachiatica* (CMW 25307) and *Calonectria pauciramosa* (CMW 5683) represent the outgroups. This is an open access article distributed under the terms of the Creative Commons Attribution License (CC BY 4.0), which permits unrestricted use, distribution, and reproduction in any medium, provided the original author and source are credited.Nam Q. Pham, Irene Barnes, ShuaiFei Chen, Thu Q. Pham, Lorenzo Lombard, Pedro W. Crous, Michael J. Wingfield

Supplementary material 2 Figure S2. Phylogenetic tree based on maximum likelihood (ML) analysis of *tef*1 sequence alignments Data type: molecular data Explanation note: Phylogenetic tree based on maximum likelihood (ML) analysis of *tef1* sequence alignments. Bootstrap value ≥ 60 % for maximum parsimony (MP) and ML analyses are indicated at the nodes. Bootstrap values lower than 60% are marked with “*” and absent are marked with “–”. Isolates representing ex–type material are marked with “T” and isolates collected in this study are highlighted in **bold**. *Calonectria brachiatica* (CMW 25307) and *Calonectria pauciramosa* (CMW 5683) represent the outgroups. This is an open access article distributed under the terms of the Creative Commons Attribution License (CC BY 4.0), which permits unrestricted use, distribution, and reproduction in any medium, provided the original author and source are credited.Nam Q. Pham, Irene Barnes, ShuaiFei Chen, Thu Q. Pham, Lorenzo Lombard, Pedro W. Crous, Michael J. Wingfield

Supplementary material 3 Figure S3. Phylogenetic tree based on maximum likelihood (ML) analysis of *tub*2 sequence alignments Data type: molecular data Explanation note: Bootstrap value ≥ 60 % for maximum parsimony (MP) and ML analyses are indicated at the nodes. Bootstrap values lower than 60 % are marked with “*” and absent are marked with “–”. Isolates representing ex–type material are marked with “T” and isolates collected in this study are highlighted in **bold**. *Calonectria brachiatica* (CMW 25307) and *Calonectria pauciramosa* (CMW 5683) represent the outgroups. This is an open access article distributed under the terms of the Creative Commons Attribution License (CC BY 4.0), which permits unrestricted use, distribution, and reproduction in any medium, provided the original author and source are credited.Nam Q. Pham, Irene Barnes, ShuaiFei Chen, Thu Q. Pham, Lorenzo Lombard, Pedro W. Crous, Michael J. Wingfield

Supplementary material 4 Figure S4. Phylogenetic tree based on maximum likelihood (ML) analysis of ITS sequence alignments Data type: molecular data Explanation note: Bootstrap value ≥ 60 % for maximum parsimony (MP) and ML analyses are indicated at the nodes. Bootstrap values lower than 60 % are marked with “*” and absent are marked with “–”. Isolates representing ex–type material are marked with “T” and isolates collected in this study are highlighted in **bold**. *Calonectria brachiatica* (CMW 25307) and *Calonectria pauciramosa* (CMW 5683) represent the outgroups. This is an open access article distributed under the terms of the Creative Commons Attribution License (CC BY 4.0), which permits unrestricted use, distribution, and reproduction in any medium, provided the original author and source are credited.Nam Q. Pham, Irene Barnes, ShuaiFei Chen, Thu Q. Pham, Lorenzo Lombard, Pedro W. Crous, Michael J. Wingfield
