# Supplementary figures and images for: New species of Cylindrocladiella from plantation soils in South-East Asia
Source: MycoKeys. 2018 Mar 15;(32):1–24. doi: 10.3897/mycokeys.32.23754 (PMC5904420; doi:10.3897/mycokeys.32.23754)

ITS

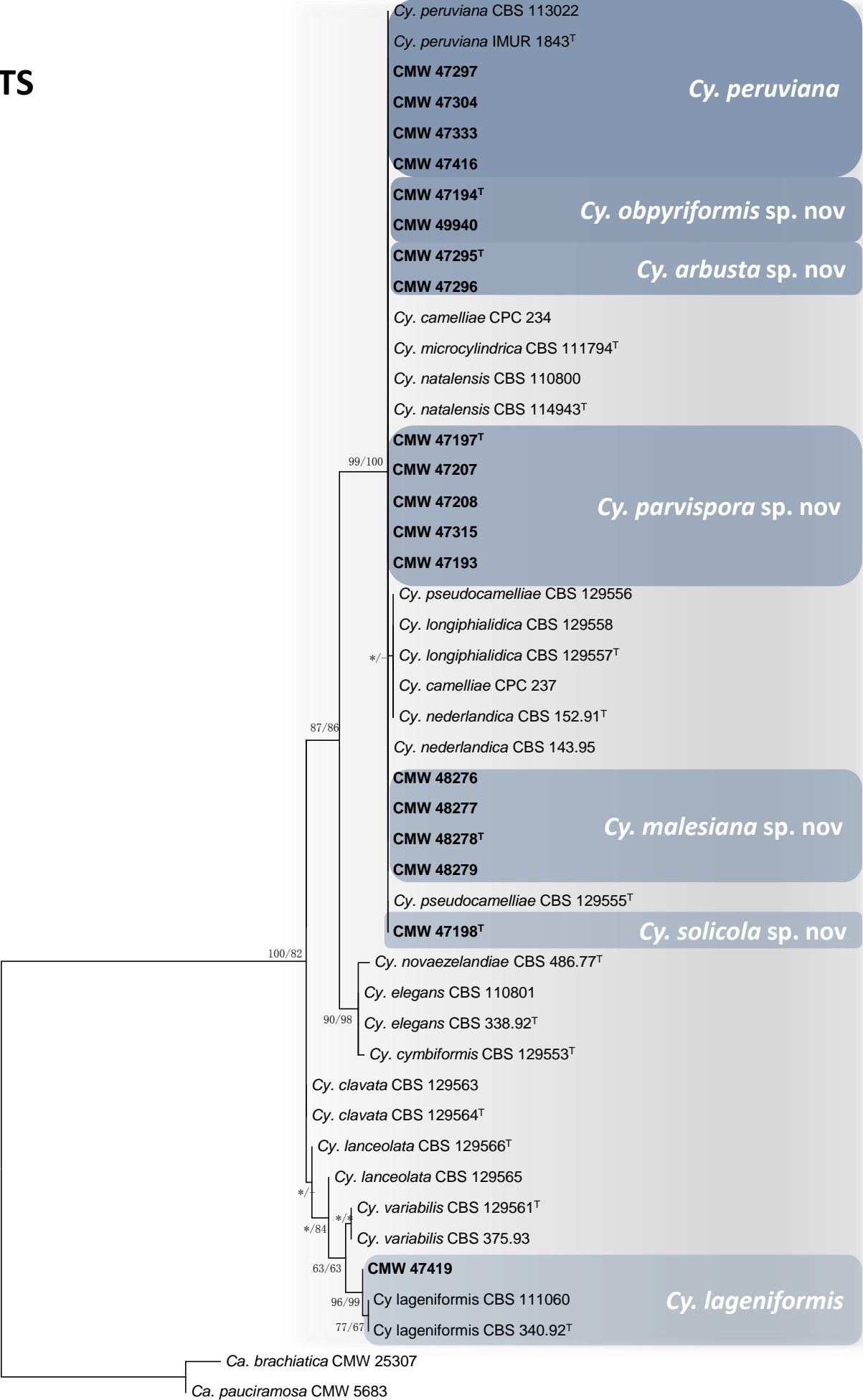

Supplement: Supplementary material 1 — Figure S1. Phylogenetic tree based on maximum likelihood (ML) analysis of his3 sequence alignments [file mycokeys-32-001-s004.pdf]
